# Association Between Oral Lichen Planus and Thyroid Disease: A Cross-Sectional Study

**DOI:** 10.3390/jcm14093106

**Published:** 2025-04-30

**Authors:** Stephanie Rodríguez-Fernández, Sonia Egido-Moreno, Sharon Rodríguez-Fernández, Joan Valls-Roca-Umbert, August Vidal-Bel, Andrés Blanco-Carrión, José López-López

**Affiliations:** 1 Department of Odontostomatology, Faculty of Medicine and Health Sciences (Dentistry), University of Barcelona, L’Hospitalet de Llobregat, 08907 Barcelona, Spain; stephroferd@gmail.com (S.R.-F.); sharonroferd@gmail.com (S.R.-F.); joan.valls@yahoo.es (J.V.-R.-U.); augustvidalbel@gmail.com (A.V.-B.); 2 Pathological Anatomy Department, University Hospital o Bellvitge, L’Hospitalet de Llobregat, 08970 Barcelona, Spain; 3 School of Medicine and Dentistry, University of Santiago de Compostela, 15782 Santiago de Compostela, Spain; andres.blanco@usc.es

**Keywords:** oral lichen planus, thyroid disease, hypothyroidism, hypertension, diabetes mellitus, dyslipidemia, anxiety, depression, association, prevalence

## Abstract

**Background:** In recent years, various authors have suggested a potential association between oral lichen planus (OLP) and thyroid disease (TD), although other studies have failed to confirm a significant relationship. The available literature presents inconsistent and sometimes contradictory findings. Additionally, other conditions—such as anxiety and depression, hypertension, diabetes mellitus, and dyslipidemia—have also been linked with OLP. **Methods:** A cross-sectional study was conducted involving 120 participants, comprising 60 patients diagnosed with OLP and 60 controls. Medical histories related to TD and other comorbidities were collected for both groups. **Results:** The prevalence of TD among the OLP patients was 20%, compared to 6.7% in the control group. The most frequently observed thyroid disorder was hypothyroidism, identified in 13.3% of the patients with OLP. Statistically, there was a moderate probability of an association between OLP and TD (*p* = 0.054). No statistically significant associations were found between OLP and hypertension (*p* = 0.378), type 2 diabetes mellitus (*p* = 0.550), dyslipidemia (*p* = 0.562), anxiety (*p* = 0.959), or depression (*p* = 0.532). **Conclusions:** Although the association between OLP and TD remains inconclusive, our findings suggest a moderate statistical probability of a relationship.

## 1. Introduction

Lichen planus (LP) is a mucocutaneous disease which can affect the oral mucosa, skin, nails, scalp, genitalia, and other mucous membranes. The most characteristic clinical feature of LP is the presence of white hyperkeratotic striae. The site most frequently affected by lichen planus is the oral mucosa, where white striae may occur as single lesions or are accompanied by erosive, atrophic, bullous, papular, or plaque lesions [1]. The prevalence in the general population is estimated to be between 1 and 2%, predominantly affecting women, most commonly from the fifth decade of life [2].

It is considered an autoimmune disease, as the immune system plays a major role in its pathogenesis. Although the exact mechanisms remain unclear, it is associated with dysregulation of the cellular immune system, in which antigen-activated CD8+ and CD4+ T cells induce epithelial cell apoptosis. Some authors suggest—although there is no clear scientific evidence—that precipitating factors such as genetic predisposition, psychological conditions (stress and anxiety), infections (such as hepatitis C), and certain systemic diseases including diabetes mellitus, thyroid disorders, dyslipidemia, and arterial hypertension may also contribute to its development [3].

The thyroid is an endocrine gland responsible for producing and secreting the hormones triiodothyronine (T3) and thyroxine (T4), which regulate metabolism, growth, and the maintenance of most bodily functions [4].

Thyroid disease (TD) includes a range of disorders caused by either excessive or insufficient production of thyroid hormones. Some of the most common thyroid conditions are hypothyroidism, hyperthyroidism, thyroid nodules, goiter, and autoimmune thyroid diseases such as Hashimoto’s thyroiditis and Graves’ disease, both caused by abnormal antibodies [5].

Currently, there is no established correlation between OLP and TD. However, immune dysregulation has been proposed as a possible explanation for a potential association. The most common autoimmune thyroid diseases are Graves’ disease (GD), which affects approximately 3% of women, and Hashimoto’s disease, which affects about 5%. The diagnosis of Hashimoto’s disease and Graves’ disease typically involves a combination of medical history, physical examination, and laboratory testing. The thyroid hormone levels (TSH, T3, and T4) are measured, along with anti-thyroid peroxidase (anti-TPO) and anti-thyroglobulin antibodies (anti-TG). Elevated TSH and low T3 and T4 levels are commonly observed in Hashimoto’s disease. In contrast, Graves’ disease is usually characterized by low TSH levels and elevated T3 and T4 levels. Additionally, thyroid-stimulating immunoglobulins (TSIs), thyrotropin receptor antibodies (TRAbs), and anti-thyroid peroxidase (anti-TPO) are assessed, as these antibodies are often elevated in Graves’ disease [6].

In recent years, various authors have proposed a possible association between OLP and TD, while others have found no significant link. Studies on this topic have produced variable and often contradictory results.

Among the evidence supporting a positive association, it has been reported that patients with Hashimoto’s thyroiditis are more likely to develop other autoimmune conditions such as OLP, as circulating thyroid antibodies may target oral keratinocytes or cross-react with proteins in keratinocyte membranes. This interaction can stimulate cytotoxic T cells to release chemokines that promote the development of OLP lesions. The correlation between these diseases may be related to immune status, suggesting shared immune triggers and pathogenic mechanisms [3]. Elevated levels of circulating antinuclear antibodies (ANAs), anti-thyroglobulin antibodies (TgAbs), and anti-thyroid microsomal autoantibodies (TPOs) in patients with OLP further support a possible association [7].

High levels of TSH (thyroid-stimulating hormone) and low levels of FT4 (free T4) have been associated with the presence of OLP, suggesting a higher prevalence of hypothyroidism in patients with OLP. However, no significant association has been found between antithyroid antibody levels and OLP [8].

Other authors have found no statistically significant differences in the incidence of OLP lesions between autoimmune and nodular thyroid disorders. They suggest that the association between OLP and hypothyroidism may be linked to an unidentified common immune process; however, this is partly contradicted by the fact that nodular thyroid disorders do not have an autoimmune etiology, although they do present immunological alterations—such as an increased number of dendritic cells, circulating lymphocytes, and inflammatory mediators like IL-6 and TNF-α—which are also involved in the pathogenesis of OLP [9,10].

At present, the scientific evidence is neither sufficiently robust nor conclusive to establish a definitive association between OLP and TD. Therefore, the aim of this study is to determine the prevalence of TD in patients with OLP and to evaluate the association between these diseases, as well as their relationship with other comorbidities.

## 2. Materials and Methods

This cross-sectional study included a total of 120 participants—60 patients diagnosed with OLP and 60 patients without a history of OLP, who served as the control group—all of whom were treated at the Dental Hospital of the University of Barcelona (HOUB). The study protocol was approved by the standing ethics committee of the Faculty of Dentistry, University of Barcelona (Comité d’Ètica de l’Hospital Odontològic, protocol code 2024-001-1). All participants provided written informed consent.

### 2.1. Data Collection

All medical histories, along with demographic information (age and sex), were collected for both groups using the GESDEN^®^ healthcare software (Gesden G5, Infomed—Henry Schein^®^, New York, NY, USA), and supplemented with medical paper records (biopsy reports) in the case of OLP patients. Recruitment and data collection were carried out from April 2024 to July 2024.

Inclusion criteria were as follows:

- Patients over 18 years of age.
- For the case group, a clinical diagnosis of OLP confirmed by pathological diagnosis was needed.
- For the control group, patients without OLP lesions, who visited HOUB for the treatment of other oral pathologies and had no history of clinically or histologically diagnosed OLP were included.

Exclusion criteria were as follows:

- Patients under 18 years of age.
- Patients diagnosed with other autoimmune diseases (e.g., systemic lupus erythematosus, Sjögren’s syndrome, rheumatoid arthritis, psoriasis, celiac disease, vitiligo, or type 1 diabetes mellitus).
- Patients with lichenoid lesions induced by drug reactions or contact with restorative materials such as amalgam.
- Pregnant women.

Participants were identified by reviewing electronic medical records, corroborated by medical reports. Medical history data included the presence of thyroid disease, specific types of TD, and associated medication, as well as a history of other comorbidities such as hypertension, type 2 diabetes mellitus, dyslipidemia, anxiety, and depression. Cases and controls were matched 1:1 by age and sex.

### 2.2. Sample Size

**Figure 1:**
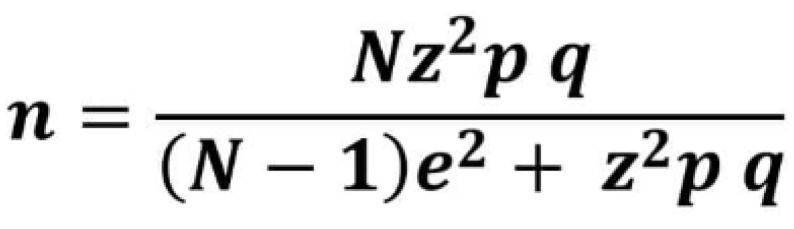
Formula for calculating the sample size. *n*: sample size; *N*: population size; *z*: statistical parameter corresponding to the desired confidence level; *e*: maximum acceptable estimation error; *p*: probability of the event occurring; *q*: probability of the event not occurring.

To calculate the sample size, we used a retrospective study by Piloni et al. [11] as a reference, as it is similar in design to the present one. In that study, a statistically significant difference was observed: the prevalence of thyroid pathology in patients with OLP was 30.26%, compared to 9.18% in the control group. We applied a formula (Figure 1) for calculating the sample size of a finite population and determined that the required sample size was 120.

### 2.3. Statistical Analysis

Data were analyzed using SPSS^®^ statistical software, version 29.0.2.0 for Windows (SPSS, Chicago, IL, USA). The prevalence of OLP, TD, and other comorbidities was evaluated using the χ^2^ test. Odds ratios (ORs) with 95% confidence intervals (CI) were calculated to assess associations between these pathologies, employing a binary logistic regression model. Statistical significance was set at *p* < 0.05.

## 3. Results

Out of a total of 120 patients, each group included 46 women (76.7%) and 14 men (23.3%), with a mean age ± standard deviation of 69.80 ± 12.58 years and an age range between 29 and 94 years. The patients with OLP were predominantly observed in the sixth and seventh decades of life. The results revealed a marked predominance of OLP among women, who accounted for 76.6% of the diagnosed cases.

Regarding the history of TD, a prevalence of 20% (*n* = 12) was observed in the patients with OLP, compared to 6.7% (*n* = 4) in the control group. This indicates that the prevalence of TD is approximately three times higher in the OLP group. According to these results, TD is statistically associated with OLP (*p* = 0.032). There is sufficient evidence to suggest that patients with OLP have a significant risk of developing a thyroid disorder compared to those without OLP. However, these results do not account for other variables.

In relation to other comorbidities, no significant differences were found between the groups. The results were not statistically significant, and there is insufficient evidence to link OLP with other pathologies such as hypertension (*p* = 0.459), type 2 diabetes mellitus (*p* = 0.624), dyslipidemia (*p* = 0.540), anxiety (*p* = 0.752) and depression (*p* = 0.408). The *p*-values indicate that no significant associations were found, as shown in Table 1.

Crosstab analyses are limited to bivariate comparisons and provide only descriptive information, without accounting for other variables that may influence the association between TD and OLP. Therefore, we employed a logistic regression model, which allows for the control of confounding factors and provides a more accurate estimate of associations. This approach enables the evaluation of how TD interacts with other variables and helps identify potential associations.

The patients with OLP were 3.3 times more likely to have a thyroid disorder compared to the individuals without OLP (OR: 3.307; 95% CI: 0.981–11.150; *p* = 0.054). This suggests a positive association between OLP and TD and supports the hypothesis that individuals with OLP may be at greater risk of developing thyroid disorders compared to those without OLP. However, this association is not entirely conclusive, as the *p*-value of 0.054 slightly exceeds the threshold for statistical significance (*p* < 0.05). The results indicate a trend toward significance, but the evidence remains inconclusive.

Regarding other comorbidities, no significant associations with OLP were found (*p* > 0.05), and the prevalence rates were similar between the cases and controls. The patients with OLP were 1.5 times more likely to have hypertension compared to the control group (OR: 1.459; 95% CI: 0.630–3.380; *p* = 0.378). The likelihood of having type 2 diabetes was lower in the OLP group compared to the control group (OR: 0.714; 95% CI: 0.236–2.158; *p* = 0.550). Dyslipidemia also showed no significant association with OLP, although the patients with OLP were 1.3 times more likely to have dyslipidemia compared to those without OLP (OR: 1.320; 95% CI: 0.516–3.373; *p* = 0.562).

Similarly, no significant associations were found between OLP and psychological disorders such as anxiety and depression. The patients with OLP were 1.0 times as likely to have anxiety (OR: 1.042; 95% CI: 0.220–4.930; *p* = 0.959) and 0.6 times as likely to have depression (OR: 0.651; 95% CI: 0.169–2.504; *p* = 0.532) compared to the control group. These associations were not statistically significant, as shown in Table 2.

A more detailed analysis of the prevalence of specific TD types was conducted. Hypothyroidism was identified as the most thyroid disorder in both groups, with a higher prevalence among the OLP patients (13.3%) compared to the control group (5%). Other thyroid disorders observed in the case group included hyperthyroidism (3.3%), thyroiditis (1.7%), and thyroid nodules (1.7%), although their overall prevalence was low. Goiter was identified in one individual from the control group (1.7%). No cases of autoimmune thyroid diseases, such as Hashimoto’s thyroiditis or Graves’ disease, were observed, as shown in Table 3.

## 4. Discussion

This study aims to determine the prevalence of thyroid disease in patients with oral lichen planus, as well as to assess the association between OLP, TD, and other comorbidities (hypertension, type 2 diabetes, dyslipidemia, anxiety, and depression).

The association between OLP and TD remains inconclusive. However, this does not preclude the existence of a direct relationship between the two conditions; a moderate association may be present, particularly when considering the χ^2^ test result (*p* = 0.032), the study’s context, and its limitations. A larger sample size may yield more statistically significant results. In this study, the prevalence of TD in patients with OLP was 20%, indicating that the risk of having a thyroid disorder is approximately three times higher than in the general population (6.7%). The most common thyroid disorder was hypothyroidism, present in 13.3% of the patients with OLP.

These findings are consistent with previous studies that have reported a positive association. Arduino et al. [9] observed a TD prevalence of 23.1% in OLP patients compared to 9.1% in a control group, with a statistically significant difference (*p* = 0.0001).

Similarly, Zhou et al. [12], in a study conducted on a Chinese population, found a TD prevalence of 72.4% in an OLP group compared to 49.4% in a control group, also with statistical significance (*p* < 0.0001).

Piloni et al. [11], in a retrospective study conducted on an Italian population, also reported a statistically significant association between OLP and thyroid disorders (OR:4.29; 95% CI:1.85–9.96; *p* = 0.0007), with a higher prevalence in the cases (30.26%) compared to the controls (9.18%). Hashimoto’s thyroiditis and hypothyroidism were identified as significant risk factors for the development of OLP lesions. The study suggested that the association between OLP and TD—particularly autoimmune conditions like Hashimoto’s thyroiditis—may be explained by a shared immune-mediated pathogenesis, indicating common autoimmune pathways between these diseases.

Similarly, in a systematic review and meta-analysis conducted by De Porras-Carrique et al. [13], the findings indicate that the global prevalence of TD in patients with OLP is 7.96%, with the risk of developing a thyroid disorder being approximately twice that of the general population. The most common thyroid disorders in patients with OLP were Hashimoto’s thyroiditis and hypothyroidism, followed by hyperthyroidism.

Our study confirms that hypothyroidism is one of the most common thyroid disorders among patients with OLP. However, we did not find evidence supporting the presence of autoimmune thyroid disorders such as Hashimoto’s thyroiditis. None of the patients were diagnosed with this condition, in contrast to findings reported in other studies.

Moreover, some studies have not found a significant association between OLP and TD. Kats et al. [14] reported no significant difference in the prevalence of TD among OLP patients (16.6%) compared to a control group (15.7%). These findings may be influenced by differences in study parameters, such as the year of completion, an insufficient sample size to accurately assess the association, the age group of the patients, or the geographical location, all of which could contribute to inconclusive results.

The relationship between OLP and TD may be multifactorial, involving chronic systemic inflammation, immunological alterations, genetic predisposition, and hormonal imbalances. The possible mechanisms of association include the increased susceptibility of individuals with one autoimmune disease to develop another, or potential interactions between the diseases themselves.

Regarding the other comorbidities analyzed, although several authors have proposed that these conditions are frequently associated with OLP and that affected patients may have a higher incidence due to various factors [15], our results do not support such a correlation. Based on the *p*-values for hypertension (*p* = 0.378), type 2 diabetes mellitus (*p* = 0.550), dyslipidemia (*p* = 0.562), anxiety (*p* = 0.959) and depression (*p* = 0.532), no statistically significant associations were observed.

Several studies have explored the association between these comorbidities and OLP. A systematic review and a meta-analysis by De Porras-Carrique et al. [16] identified a potentially increased risk of hypertension in patients with OLP (OR:1.28; 95% CI: 1.01–1.63; *p* = 0.04). Similarly, Dave et al. [17] found that patients with OLP are more likely to develop type 2 diabetes compared to the general population (OR:2.8; 95% CI: 1.2–6.3; *p* = 0.013).

Li et al. [18] also reported a positive association between OLP and dyslipidemia (OR:4.52; 95% CI: 2.49–8.18; *p* < 0.001). Furthermore, Vallejo et al. [19] observed that patients with OLP have significantly higher levels of anxiety (OR:2.8; 95% CI: 1.0–7.4; *p* < 0.001) and depression (OR:4.4; 95% CI: 1.8–10.6; *p* < 0.001) compared to control groups, suggesting that these psychological disorders may play a role in the development of OLP.

The main limitation could be the differentiation between OLP and OLL, which, sometimes, can be a challenge [20]. Despite the classification proposed by van der Meij and van der Waal [21], it has been recognized that there is no pathognomonic histopathological pattern or no specific data that allow for distinguishing its main subtypes. Another limitation of the study was the design and the limited information available in the patients’ medical records presented to us, in order to establish whether OLP precedes TD or vice versa. Therefore, a causal relationship could not be determined.

## 5. Conclusions

The prevalence of thyroid disease in patients with oral lichen planus is 20%, with the risk of having a thyroid disorder approximately three times higher than in the general population. The most common thyroid disorder is hypothyroidism, present in 13.3% of patients with OLP. Although the association between OLP and TD is still unclear, our findings suggest a moderate statistical likelihood of such a relationship. The other comorbidities analyzed—arterial hypertension, type 2 diabetes mellitus, dyslipidemia, anxiety, and depression—did not show a significant association with OLP.

## Figures and Tables

**Table 1:** Prevalence of TD and other comorbidities in OLP patients and controls.

	OLP *n* (%)	Controls *n* (%)	*p*-Value
Age	69.80 ± 12.58	69.80 ± 12.58	1000
Sex			1000
Woman	46 (76.7%)	46 (76.7%)	
Man	14 (23.3%)	14 (23.3%)	
Thyroid disease	12 (20%)	4 (6.7%)	0.032 *
Other comorbidities			
Hypertension	27 (45%)	23 (38.3%)	0.459
Type 2 diabetes mellitus	9 (15%)	11 (18.3%)	0.624
Dyslipidemia	18 (30%)	15 (25%)	0.540
Anxiety	5 (8.3%)	6 (10%)	0.752
Depression	6 (10%)	9 (15%)	0.408

OLP: oral lichen planus; * significance at *p* < 0.05.

**Table 2:** Association of TD and other comorbidities in OLP patients and control group.

	OR	*p*-Value	95% CI	
			Inferior	Superior
Age	1.006	0.701	0.974	1.039
Sex	0.974	0.953	0.402	2.361
Thyroid disease	3.307	0.054	0.981	11.150
Hypertension	1.459	0.378	0.630	3.380
Type 2 diabetes mellitus	0.714	0.550	0.236	2.158
Dyslipidemia	1.320	0.562	0.516	3.373
Anxiety	1.042	0.959	0.220	4.930
Depression	0.651	0.532	0.169	2.504

Binary logistic regression. OR: odds ratio; CI: 95% confidence interval.

**Table 3:** Prevalence of most common thyroid disorders in OLP patients and controls.

		OLP *n* (%)	Controls *n* (%)	Total *n* (%)
**Thyroid disease**	Hypothyroidism	8 (13.3%)	3 (5.0%)	11 (9.2%)
	Hyperthyroidism	2 (3.3%)	0 (0.0%)	2 (1.7%)
	Goiter	0 (0.0%)	1 (1.7%)	1 (0.8%)
	Thyroiditis	1 (1.7%)	0 (0.0%)	1 (0.8%)
	Graves’ disease	0 (0.0%)	0 (0.0%)	0 (0.0%)
	Hashimoto’s thyroiditis	0 (0.0%)	0 (0.0%)	0 (0.0%)
	Thyroid nodules	1 (1.7%)	0 (0.0%)	1 (0.8%)
	Absence of disease	48 (80%)	56 (93.3%)	104 (86.7%)
**Total *n* (%)**		60 (100%)	60 (100%)	120 (100%)

OLP: oral lichen planus.

## Data Availability

The original contributions presented in this study are included in the article. Further inquiries can be directed to the corresponding authors.
